# Tomatidine suppresses inflammation in primary articular chondrocytes and attenuates cartilage degradation in osteoarthritic rats

**DOI:** 10.18632/aging.103222

**Published:** 2020-07-05

**Authors:** Xiangyu Chu, Tao Yu, Xiaojian Huang, Yang Xi, Bowei Ni, Rui Zhang, Hongbo You

**Affiliations:** 1 Department of Orthopedics, Tongji Hospital, Tongji Medical College, Huazhong University of Science and Technology, Wuhan 430030, Hubei, China; 2 Department of Orthopedic Surgery, Tongji Hospital, Tongji University School of Medicine, Shanghai 200065, China

**Keywords:** tomatidine, osteoarthritis, interleukin-1β, chondrocyte, NF- κB

## Abstract

In this study, we investigated whether the anti-inflammatory effects of tomatidine alleviate osteoarthritis (OA)-related pathology in primary articular chondrocytes and a rat OA model. STITCH database analysis identified 22 tomatidine-target genes that were enriched in 78 Kyoto Encyclopedia of Genes and Genomes (KEGG) pathways. Moreover,39 of the 105 OA-related KEGG pathways were related to tomatidine-target genes. The top two OA-related KEGG pathways with tomatidine-target genes were the MAPK and neutrophin signaling pathways. Pretreating primary chondrocytes with tomatidine suppressed interleukin-1β (IL-1β)-induced expression of iNOS, COX-2, MMP1, MMP3, MMP13, and ADAMTS-5. Tomatidine also suppressed IL-1β-induced degradation of collagen-II and aggrecan proteins by inhibiting NF-κB and MAPK signaling. In a rat OA model, histological and immunohistochemical analyses showed significantly less cartilage degeneration in thetibiofemoral joints of rats treated for 12 weeks with tomatidine after OA induction (experimental group) than in untreated OA group rats. However, micro-computed tomography (μ-CT) showed that tomatidine did not affect remodeling of the subchondral bone at the tibial plateau. These data shows that tomatidine suppresses IL-1β-induced inflammation in primary chondrocytes by inhibiting the NF-κB and MAPK signaling pathways, and protects against cartilage destruction in a rat OA model.

## INTRODUCTION

Osteoarthritis (OA) is a degenerative joint disease associated with chronic pain and disability; it is characterized by articular cartilage breakdown, synovial inflammation, and bone hypertrophy [1]. The constantly increasing numbers of OA patients worldwide incurs an expenditure of 1.0% to 2.5% Gross Domestic Product (GDP) in developed countries [2, 3]. OA is a progressive joint disease without any effective curative treatment [4–6]. Therefore, it is of paramount importance to identify the underlying pathways involved in OA pathology and discover new therapies that can improve the quality of life for patients with OA.

The hallmark of OA is the imbalance between pro-inflammatory and anti-inflammatory cytokines [7]. The most critical pro-inflammatory cytokine involved in OA pathogenesis is interleukin-1β (IL-1β), which downregulates key extracellular matrix (ECM) proteins, type-II collagen and aggrecan, and upregulates matrix metalloproteinases (MMPs) and ADAMTS-5, which breakdown the cartilage tissue [8, 9]. Therefore, anti-inflammatory drugs are potential therapeutic candidates against OA.

Tomatidine, the major glycoalkaloid produced by the tomato plant, is associated with anti-apoptotic, anti-inflammatory, anti-bacterial, and anti-cancer properties [10–14]. Tomatidine suppresses inflammation in LPS-stimulated murine macrophages by inhibiting the NF-κB and JNK signaling pathways [15]. However, the therapeutic effects of tomatidine in OA are unknown. Therefore, in this study, we investigated the effects of tomatidine on IL-1β-induced inflammation in primary articular chondrocytes and cartilage degeneration in the rat OA model.

## RESULTS

### Bioinformatics analysis of tomatidine-target genes and OA-related KEGG pathways

**Figure 1:**
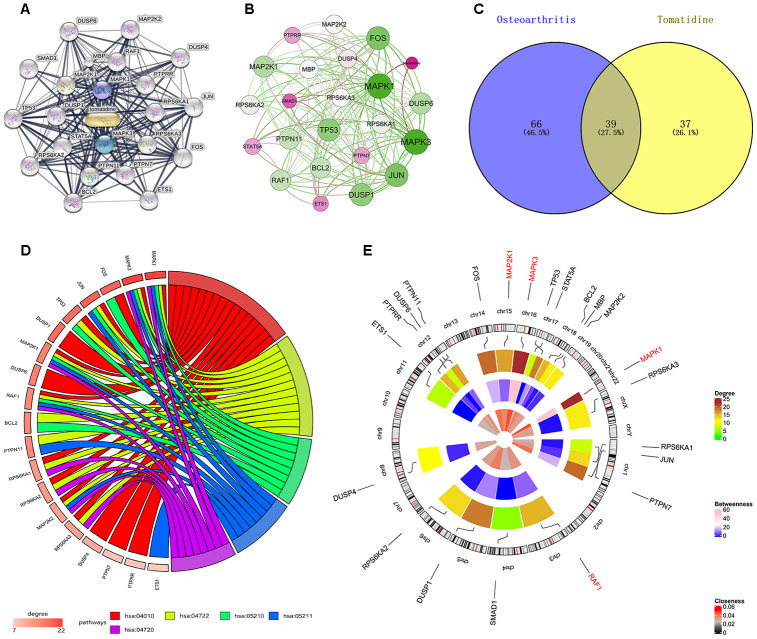
**Identification of tomatidine-target genes and the common KEGG pathways of OA-related and tomatidine-target genes.** (**A**) Interaction network of 22 tomatidine-target genes based on STITCH database analysis. (**B**) *MAPK1, MAPK3, JUN, DUSP1, FOS*, and *TP53* have the highest weights in the interaction network constructed using Gephi. (**C**) KEGG pathway analyses shows 105 OA-related and 76 tomatidine-target gene-related KEGG pathways. Among these, 39 (27.5%) KEGG pathways are common to both OA and tomatidine-target genes. (**D**) Gene enrichment analyses show that MAPK1, MAP2K1, MAPK3, and RAF1 are involved in all the top five KEGG pathways. The top three genes with highest degree are MAPK1, MAPK3, and FOS. (**E**) The circular visualization shows chromosomal positions and connectivity of tomatidine-target genes. The names of the tomatidine-target genes are shown in the outer circle, which represents chromosomes. The lines from each gene represent specific chromosomal locations. colors show Different values of degree, betweenness and closeness are shown in different colors. The three hub genes are shown in red.

The interaction network shows 22 tomatidine-target genes belonging to three shells based on the STITCH database analysis (Figure 1A). The first shell contained two genes, *MAPK1* and *MAPK3*; the second shell contained 10 genes, *RAF1*, *PTPRR*, *RPS6KA1*, *RPS6KA3*, *PTPN7*, *PTPN11*, *STAT5A*, *DUSP1*, *MAP2K1*, and *MBP*; and the third shell contained 10 genes, *MAP2K2*, *DUSP4*, *JUN*, *FOS*, *3TS1*, *BCL2*, *RPS6KA2*, *TP53*, *SMAD1*, and *DUSP6*. The weight of *MAPK1* was the highest according to the interaction network analysis of the tomatidine-target genes (Figure 1B). DAVID database analysis identified 78 KEGG pathways (p< 0.05) with tomatidine-target genes, whereas, miRWalk2.0 database analysis showed 105 human OA-related KEGG pathways [16]. As shown in the Venn diagram, 39 OA-related KEGG pathways contained tomatidine-target genes (Figure 1C). The top five OA-related KEGG pathways with tomatidine-target genes include MAPK signaling, neurotrophin signaling, colorectal cancer, renal cell carcinoma, and long-term potentiation pathways (Table 1).

**Table 1:** Top five KEGG pathway and involved genes.

**Term**	**KEGG Pathway**	**Tomatidine-target Genes**	***P*-value**
hsa04010	MAPK signaling pathway	*PTPN7, MAP2K1, MAP2K2, TP53, PTPRR, RAF1, DUSP4, MAPK1, FOS, RPS6KA3, RPS6KA1, DUSP1, RPS6KA2, JUN, MAPK3, DUSP6*	2.6E-18
hsa04722	Neurotrophin signaling pathway	*MAPK1, RPS6KA3, RPS6KA1, MAP2K1, RPS6KA2, MAP2K2, JUN, BCL2, MAPK3, TP53, RAF1, PTPN11*	4.2E-15
hsa05210	Colorectal cancer	*MAPK1, FOS, MAP2K1, JUN, BCL2, MAPK3, TP53, RAF1*	2.4E-10
hsa05211	Renal cell carcinoma	*MAPK1, MAP2K1, ETS1, MAP2K2, JUN, MAPK3, RAF1, PTPN11*	3.8E-10
hsa04720	Long-term potentiation	*MAPK1, RPS6KA3, RPS6KA1, MAP2K1, RPS6KA2, MAP2K2, MAPK3, RAF1*	3.8E-10

### Identification of the hub genes and KEGG pathways related to tomatidine-target genes

**Figure 2:**
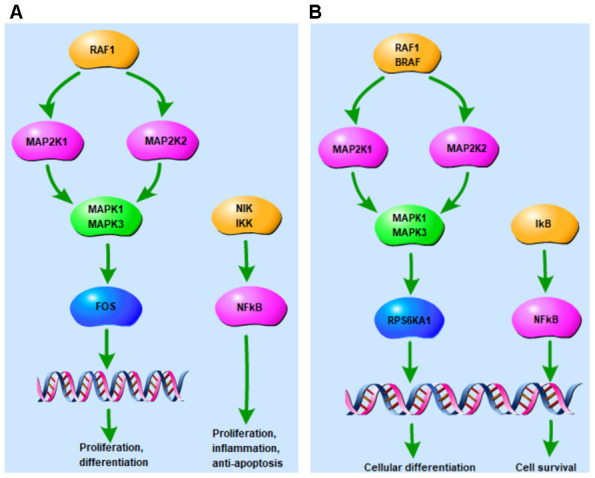
**Tomatidine-target genes associated with the top two KEGG pathways.** (**A**) The tomatidine-target genes that are part of the MAPK signaling pathway, including *MAPK1* and *MAPK3* are associated with proliferation, differentiation, cell survival or anti-apoptotic, and inflammation. (**B**) Tomatidine-target genes that are part of the Neurotrophin signaling pathway, including *MAPK1* and *MAPK3* are associated with cellular differentiation, cell survival, and retrograde transport.

Figure 1D shows the enrichment information of the 22 tomatidine-target genes involved in the top five OA-related KEGG pathways. Among these, *MAPK1, MAP2K1, MAPK3*, and *RAF1* are involved in all the top five KEGG pathways and are considered as hub genes. Figure 1E represents the circular diagrammatic representation highlighting the chromosomal positions and connectivity of the tomatidine-target genes. Among the 22 tomatidine-target genes, *MAPK1* shows the greatest degree, betweenness, and closeness centrality. The top 2 shared KEGG pathways with highest p values are associated with inflammation, proliferation, differentiation, pro-survival, and retrograde signaling via MAPK and NF- κB signaling pathways (Figure 2A, 2B).

### Low-dose tomatidine does not affect viability of IL-1β-treated primary chondrocytes

**Figure 3:**
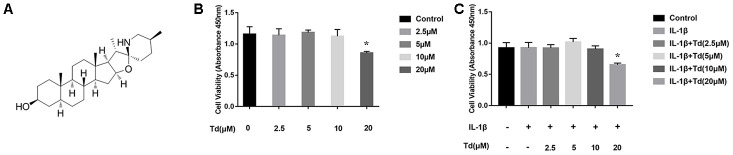
**Effects of tomatidine on the viability of primary chondrocytes.** (**A**) Chemical structure of tomatidine. (**B**, **C**) CCK-8 assay shows the viability of primary chondrocytes treated for 24 h with (**B**) 2.5, 5, 10 or 20 μM tomatidine alone or in combination with 10 ng/mlIL-1β (**C**). DMSO was used as control. As shown, treatments with 2.5, 5, or 10μM tomatidine or 10ng/mlIL-1β do not affect cell viability. Viability of primary chondrocytes is significantly affected by treatment with 20μM tomatidine in presence or absence of 10ng/mlIL-1β. The values are shown as means ± SD. *p < 0.05compared with the control group.

CCK-8 assay showed that pretreatment of primary chondrocytes with 2.5, 5, or 10μM tomatidine followed by 10ng/ml IL-1β did not affect cell viability compared to the controls (P > 0.05; Figure 3A–3C). However, pretreatment with 20μM tomatidine significantly decreased the viability of primary chondrocytes (P < 0.05; Figure 3B, 3C). We chose 2.5, 5, and 10μM tomatidine doses for subsequent experiments.

### Tomatidine inhibits IL-1β-induced iNOS and COX-2 expression in primary chondrocytes

**Figure 4:**
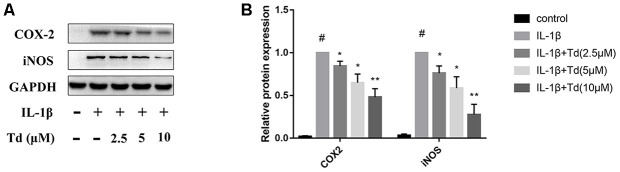
**Tomatidine inhibits IL-1β-induced iNOS and COX-2 expression in primary chondrocytes.** (**A**) Representative western blot images and (**B**) Histogram plots show the levels of iNOS and COX-2 proteins relative to GAPDH (internal control) levels in primary chondrocytes treated for 24 h with 2.5, 5, or 10μM tomatidine alone or in combination with 10ng/mlIL-1β. DMSO was used as control. The values are shown as means ± SD of triplicate experiments. #p < 0.05 compared with the control group; *p < 0.05 and **p < 0.01 compared with the IL-1β group.

We analyzed the effects of tomatidine pre-treatment on the IL-1β-induced expression of iNOS and COX-2 in primary chondrocytes. IL-1β-treatedprimary chondrocytes showed significantly higher levels of iNOS and COX-2 proteins compared to the blank control, but, pre-treatment with 2.5, 5.0 and 10μM tomatidine significantly reduced iNOS and COX-2 expression in a concentration-dependent manner (Figure 4A, 4B). These data demonstrate that tomatidine inhibits IL-1β-induced iNOS and COX-2 expression in primary chondrocytes.

### Tomatidine inhibits IL-1β induced MMPs and ADAMTS-5 in chondrocytes

**Figure 5:**
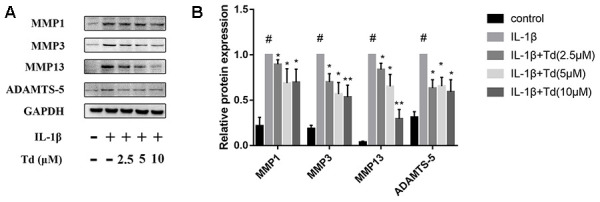
**Tomatidine inhibits IL-1β induced MMPs and ADAMTS-5 in chondrocytes.** (**A**) Representative western blot images and (**B**) Histogram plots show the levels of MMP1, MMP3, MMP13 and ADAMTS-5proteins relative to GAPDH (internal control) levels in primary chondrocytes treated for 24 h with 2.5, 5, or 10μM tomatidine alone or in combination with 10ng/mlIL-1β. DMSO was used as control. The values are shown as means ± SD of triplicate experiments. #p < 0.05 compared with the control group. *p < 0.05 and **p < 0.01 compared with the IL-1β group.

Next, we analyzed the effects of tomatidine on IL-1β-induced expression of MMPs (MMP1, MMP3, and MMP13) and ADAMTS-5 in primary chondrocytes. IL-1β-treatedprimary chondrocytes showed significantly higher expression of MMPs and ADAMTS-5 compared to the blank controls, but, tomatidine significantly reduced the expression of MMPs and ADAMTS-5 in a concentration-dependent manner (Figure 5A, 5B). These findings show that tomatidine protects against OA progression by suppressing the expression of MMPs and ADAMTS-5 in primary chondrocytes.

### Tomatidine suppresses IL-1β induced degradation of aggrecan and collagen-II in primary chondrocytes

**Figure 6:**
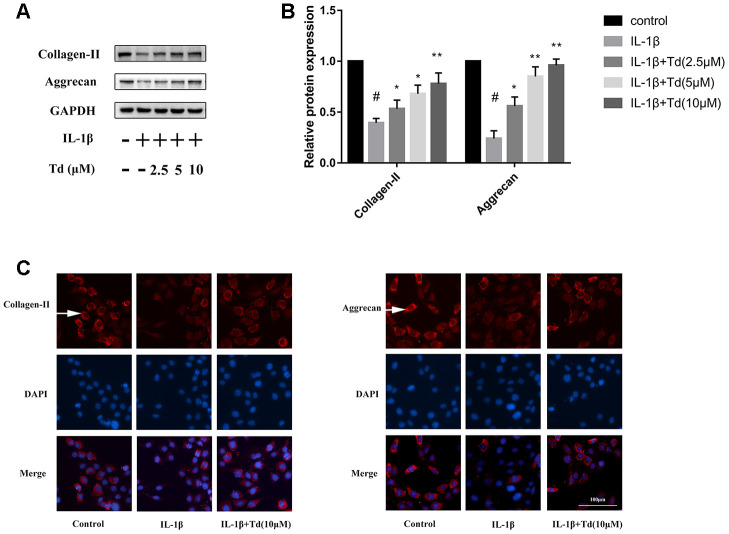
**Tomatidine suppresses IL-1β induced degradation of aggrecan and collagen-II in chondrocytes.** (**A**) Representative western blot images and (**B**) Histogram plot shows the levels of aggrecan and collagen-II proteins relative to GAPDH (internal control) levels in the primary chondrocytes treated for 24 h with 2.5, 5, or 10μM tomatidine alone or in combination with 10ng/mlIL-1β. DMSO was used as control. (**C**) Immunofluorescence staining images show aggrecan and collagen-II expression in the primary chondrocytes treated for 24 h with 2.5, 5, or 10μM tomatidine alone or in combination with 10ng/mlIL-1β. The nuclei are stained with DAPI. The white arrows indicate the expression of aggrecan and collagen-II. The values are shown as means ± SD of triplicate experiments. #p < 0.05 compared with the control group; *p < 0.05 and **p < 0.01 compared with the IL-1β group.

IL-1β-treatedprimary chondrocytes showed significantly higher degradation of ECM proteins, aggrecan and collagen-II, compared with the blank controls based on western blotting and immunofluorescence staining assays (Figure 6A–6C). However, tomatidine significantly reduced the degradation of aggrecan and collagen-II in the IL-1β-treated primary chondrocytes in a concentration dependent manner (Figure 6A–6C).

### Tomatidine suppresses IL-1β induced activation of NF-κB in primary chondrocytes

**Figure 7:**
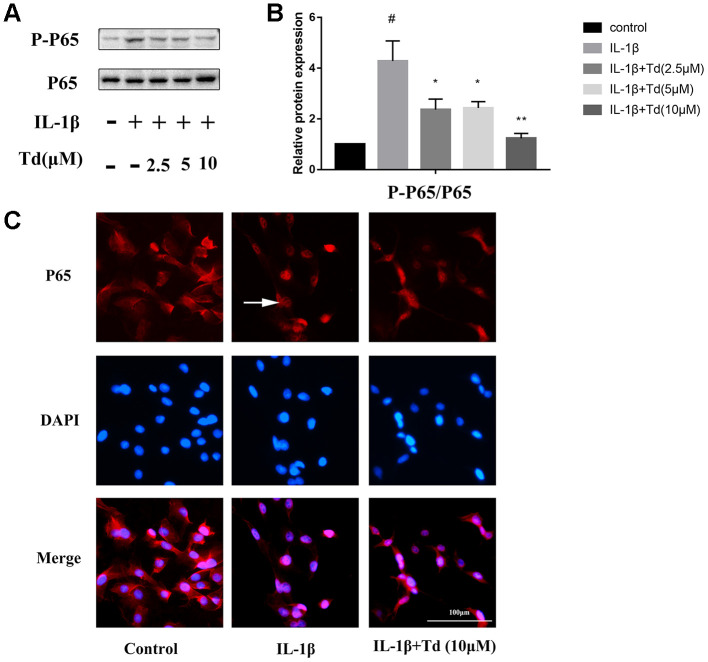
**Tomatidine suppresses IL-1β-induced NF-κB activation in primary chondrocytes.** (**A**) Representative western blot images and (**B**) Histogram plots show the levels of phospho-P65 and P65 proteins relative to GAPDH (internal control) levels in the primary chondrocytes treated for 30 mins with 2.5, 5, or 10μM tomatidine alone or in combination with 10 ng/mlIL-1β. DMSO was used as control. (**C**) Immunofluorescence staining images show P65 localization in primary chondrocytes treated for 30 mins with 2.5, 5, or 10μM tomatidine alone or in combination with 10ng/mlIL-1β. The white arrow indicates nuclear translocation of P65. The values are shown as means ± SD of triplicate experiments. #p < 0.05 compared with the control group; *p < 0.05 and **p < 0.01 compared with the IL-1β group.

We analyzed the effects of tomatidine on IL-1β-induced activation of NF-κB in primary chondrocytes. IL-1β-treated primary chondrocytes showed significantly higher levels of phospho-P65 compared to the blank controls, but, pre-treatment with tomatidine significantly reduced phospho-P65 levels in a concentration-dependent manner (Figure 7A, 7B; P < 0.05). Immunofluorescence assay showed that tomatidine inhibits nuclear translocation of P65 inIL-1β-treated primary chondrocytes in a concentration dependent manner (Figure 7C). These results demonstrate that tomatidine suppresses activation of the NF-κB in IL-1β-treated primary chondrocytes.

### Tomatidine suppresses IL-1β-induced activation of MAPK signaling pathway in primary chondrocytes

**Figure 8:**
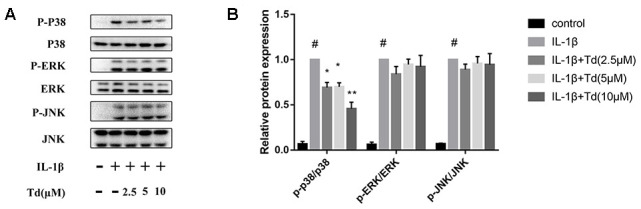
**Tomatidine inhibits IL-1β-induced MAPK activation in primary chondrocytes.** (**A**) Representative western blot images and (**B**) Histogram plot shows the levels of phospho-P38, P38, phospho-ERK, ERK, phospho-JNK, and JNK proteins relative to GAPDH (internal control) levels in the primary chondrocytes treated for 30 mins with 2.5, 5, or 10μM tomatidine alone or in combination with 10ng/mlIL-1β. DMSO was used as control. The values are means ± SD of triplicate experiments. #p < 0.05 compared with the control group. *p < 0.05 and **p < 0.01 compared with the IL-1β group.

Next, we analyzed the effects of tomatidine on the activation status of the MAPK signaling pathway in IL-1β-treated primary chondrocytes. As shown in Figure 8A, 8B, IL-1β-treated primary chondrocytes showed significantly higher levels of phospho-P38, phospho-ERK, and phospho-JNK compared to the blank controls, but, tomatidine suppressed P38 activation in a concentration-dependent manner (Figure 8A, 8B; P < 0.05). These data demonstrate that tomatidine reduces inflammation in primary chondrocytes by inhibiting the activation of p38 MAPK signaling pathway.

### Tomatidine ameliorates OA progression in the rat OA model

**Figure 9:**
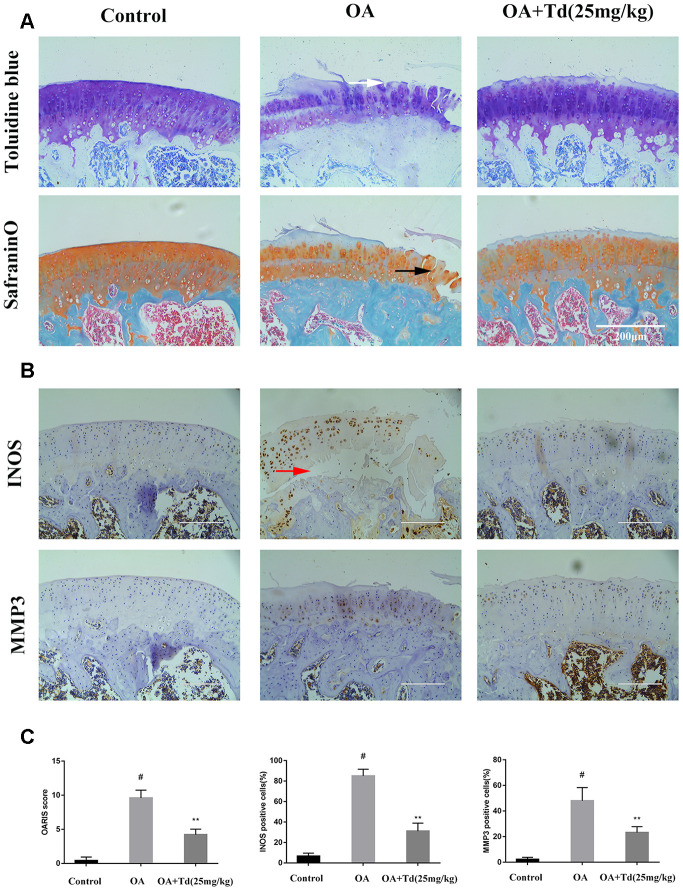
**Tomatidine ameliorates OA progression in the rat OA model. Sprague Dawley rats (n=5/group) were randomly divided into blank control, OA, and experimental groups.** The experimental group rats were fed a diet that included 25mg/kg/day tomatidine. The rats were maintained in these groups for 12 weeks and then euthanized and their tibiofemoral joints were obtained and processed (**A**) Representative images show safranin O-fast green(S-O) and toluidine blue stained sections of tibiofemoral joints from blank control, OA and experimental group rats. The vertical fissures (black arrow), surface discontinuity (white arrow) and delamination (red arrow) are indicated in the relevant samples as shown. (**B**) Representative immunohistochemial stained images show the expression of iNOS and MMP3 proteins in tibiofemoral joint sections from blank control, OA and experimental group rats. (**C**) The OA grades of blank control, OA and experimental group rats at 12 weeks according to the Osteoarthritis Research Society International (OARSI) scores are shown. The scoring was performed in a blinded manner. The iNOS and MMP3 positive cells were counted in each tibiofemoral joint section from blank control, OA and experimental group rats and quantified using the Image-J software. #p < 0.05 compared with the control group. *p < 0.05 and **p < 0.01 compared with the OA group.

Next, we analyzed the effects of tomatidine on the progression of OA in the rat model. Histological analysis showed that damage to the articular cartilage was significantly reduced in the experimental group rats (OA model rats treated with tomatidine) compared to the OA group (Figure 9A). Furthermore, immunohistochemical staining showed that tomatidine significantly reduced INOS and MMP3levels in the articular cartilage of experimental group rats compared to the OA group (Figure 9B, 9C). This demonstrates that tomatidine ameliorates OA progression in the rat OA model.

### μ-CT evaluation of the effects of tomatidine treatment on remodeling of the subchondral bone in OA model rats

**Figure 10:**
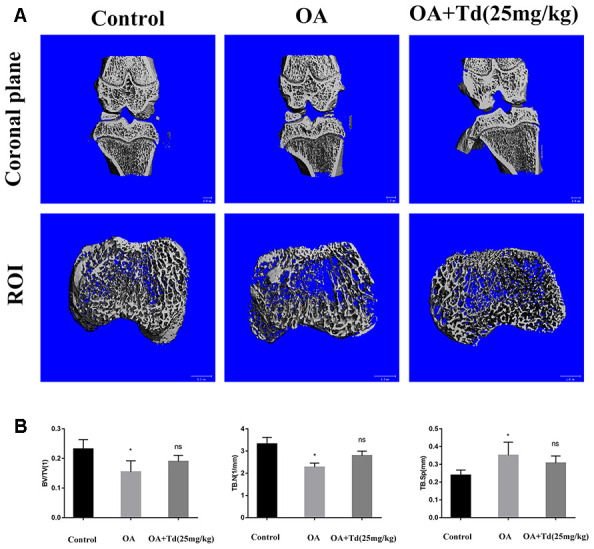
**μ-CT evaluation of the subchondral bone at the tibial plateau in the OA model rats.** (**A**) Representative images show the 3D μ-CT coronal views of the right knee and axial view of ROI from blank control, OA and experimental group rats. (**B**) Histogram plots show the analysis of trabecular structure analysis, including bone volume/tissue volume (BV/TV), trabecular number (Tb. N) and trabecular separation (Tb. Sp)in the blank control, OA and experimental group rats. *p < 0.05 compared with the control group; ns: not significant compared with the OA group.

We performed μ-CT to determine the effects of tomatidine on the subchondral bone remodeling in the OA model rats. The OA group rats showed decreased BV/TV and trabecular numbers and increased trabecular separation in the subchondral bones compared to the blank control group (Figure 10A, 10B; P < 0.05). However, the BV/TV, trabecular numbers and trabecular separation parameters were similar for both OA and tomatidine-treated experimental group rats (Figure 10A, 10B; P > 0.05). These results show that tomatidine does not affect subchondral bone remodeling in OA model rats.

## DISCUSSION

Our study demonstrates that tomatidine inhibits IL-1β-induced expression of iNOS, COX-2, and MMPs, and degradation of aggrecan and collagen-II in primary chondrocytes by suppressing the activation of NF- κB and MAPK signaling pathways. These results show that tomatidine sustains the levels of ECM proteins by suppressing the expression of proteins involved in inflammation and degradation of ECM proteins. Moreover, tomatidine reduces cartilage degeneration in the rat OA model. These findings reveal that tomatidine is a promising drug for OA treatment.

IL-1β is a major pro-inflammatory cytokine that is involved in OA pathology by inducing inflammatory responses and catabolic effects [9, 17–19]. IL-1β promotes the expression of MMPs, which are involved in the degradation of cartilage components [20]. IL-1β also induces ADAMTS-5, which promotes breakdown of aggrecan [21, 22]. IL-1β stimulates the expression of iNOS and COX-2, which promote the release of well known inflammatory mediators, NO and PGE2 [23, 24]. Several studies suggest that inhibition of these pro-inflammatory mediators hinders OA progression [19, 20].

In this study, we hypothesized that the anti-inflammatory properties of tomatidine will delay or abrogate OA progression in the primary chondrocytes and cartilage tissue. Chiu et al. reported that tomatidine inhibits the inflammatory response in LPS-stimulated mouse macrophages by suppressing the NF-κB and JNK signaling pathways [15]. The activation of NF-κB signaling pathway is implicated in the onset and progression of OA [25]. NF-κB pathway also regulates the expression of iNOS, COX-2, and MMPs [26, 27]. Therefore, targeted inhibition of the NF- κB signaling pathway is a potential therapy for patients with OA. We demonstrate that tomatidine significantly inhibits IL-1β-induced expression of inflammatory factors by suppressing NF-κB activation. MAPK signaling is another key pathway involved in OA pathogenesis [28]. The OA chondrocytes demonstrate significantly higher levels of phospho-p38, phospho-JNK, and phospho-ERK compared to normal chondrocytes [29]. Our study demonstrates that tomatidine blocks IL-1β-stimulated MAPK pathway activation in primary articular chondrocytes. Overall, our study demonstrates that tomatidine exerts anti-inflammatory effects on IL-1β-stimulated chondrocytes by suppressing the NF- κB and MAPK signaling pathways.

Our study has several limitations. Firstly, it is not clear if tomatidine inhibits NF- κB and MAPK pathways directly or indirectly through mediators. Secondly, a higher concentration of tomatidine does not demonstrate anti-inflammatory and cell survival effects, probably because it exceeds the optimal dosage.

## CONCLUSIONS

**Figure 11:**
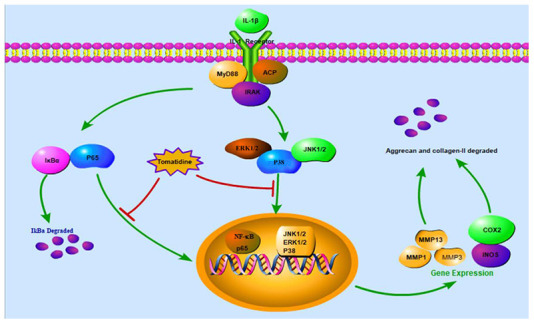
**The anti-inflammatory mechanism of action of tomatidine in primary articular chondrocytes and the OA model rats.** The schematic diagram shows that tomatidine suppresses IL-1β-induced expression of iNOS, COX-2, MMPs and ADAMTS-5, and degradation of aggrecan and collagen-II by inhibiting the NF-κB and MAPK pathways.

In summary, our study shows that tomatidine inhibits IL-1β-stimulated inflammation in primary chondrocytes by suppressing the NF-κB and MAPK signaling pathways (Figure 11). Moreover, tomatidine decreases the articular cartilage pathology in the rat OA model. These findings suggest that tomatidine is a potential treatment for OA, which needs to be ascertained by further investigations.

## MATERIALS AND METHODS

### Identification of tomatidine-target genes, their interaction network and the OA-related KEGG pathways containing tomatidine-target genes

We identified and constructed an interaction network between tomatidine-target genes in first, second, and third shells using the Search Tool for Interacting Chemicals (STITCH) database [30]. Tomatidine was imported into the STITCH, and the predicted garget-genes were abtained with default settings. Then, we calculated the degree, betweenness, and closeness of the tomatidine-related genes in the network using Cytoscape 3.7.2 [31]. Degree Centrality represents the number of links a node has with other nodes in the network. Closeness Centrality is a measure of the centrality of a node in a network, that is, more central the node is, the closer it is to other nodes in the network. Betweenness Centrality measures how important a node is based on all the number of shorter paths between a pair of nodes. We used the miRWalk2.0 database to retrieve KEGG pathways involved in human OA [16]. We used the Database for Annotation, Visualization, and Integrated Discovery (DAVID) to identify KEGG pathways enriched (p<0.05) with the tomatidine-target genes [32, 33]. The Venn Diagram (Venny 2.1, http://bioinfogp.cnb.csic.es/tools/venny/index.html) was used to show the KEGG pathways that are common to OA and tomatidine-target genes.

### Identification of the hub genes and KEGG pathways related to tomatidine-target genes

The GO plots were used to present the enrichment information of the top five KEGG pathways [34]. The genes in the top five KEGG pathways were considered as the hub genes. The circlize R package was used to determine the centrality of all the tomatidine-targeted genes in the network [35]. The top 5 KEGG pathways with the smallest p values were selected and their relationship with the tomatidine-target genes was established using the Pathway Builder Tool 20 (www.proteinlounge.com).

### Chemicals and reagents

Tomatidine (purity>98%) was purchased from MedChem Express (Princeton, NJ). Collagenase type II, recombinant rat IL-1β, and dimethylsulfoxide (DMSO) were obtained from Sigma Chemical Co. (St. Louis, MO, USA). Cell Counting Kit-8 (CCK-8) was obtained from Boster (Wuhan, China). The primary antibody against aggrecan was purchased from Abcam (Cambridge, UK). The antibodies against type-II collagen, iNOS, and MMP13 were purchased from Santa Cruz Biotechnology (Santa Cruz, CA, USA), whereas, antibodies against COX2, MMP3, P38, P-P38, ERK, P-ERK, JNK, P-JNK, P65, P-P65 were obtained from the Cell Signaling Technology Incorporated (Beverly, MA, USA). The antibodies against GAPDH, ADAMTS-5 and MMP1 were obtained from Boster (Wuhan, China). Fetal bovine serum (FBS), bovine serum albumin (BSA), and Dulbecco’s modified Eagle’ ’s medium F12 (DMEM/F12) were purchased from Gibco (NY, USA).

### Primary chondrocyte isolation and culture

The primary chondrocytes were isolated as described previously [36]. Briefly, the articular cartilage was obtained from the knees of male Sprague-Dawley rats, cut into 1 mm^3^pieces, and digested with trypsin for 30 min at 37°C. Then, the primary chondrocytes were obtained by digestion with 0.2% collagenase II for 4-6 h at 37°C. The primary chondrocytes were cultured in DMEM/F12 medium supplemented with 10% FBS and 1% penicillin/streptomycin at 37°C and 5% CO2. The medium was changed every two days. The primary chondrocytes from 2^nd^ and 3^rd^passages were used for further experiments.

### Cell viability assay

Tomatidine cytotoxicity was analyzed using the CCK-8 assay according to manufacturer’s instructions. Briefly, the primary chondrocytes were seeded in a 96-well plate (5 × 10^3^/well) for 24h, pre-treated with different concentrations of tomatidine (2.5, 5, 10 or 20μM) for 2 h then treated with10ng/mlIL-1β for 24h. Primary chondrocytes treated with DMSO, tomatidine or IL-1β alone were used as controls. Subsequently, the cells were incubated with 10μl CCK-8 solution for 1h at 37°C. The absorbance was measured at 450nm using a plate reader (Bio-Rad, Richmond, CA, USA).

### Western blotting

The primary chondrocytes were seededin a 6-well plate at 37°C and 5% CO2 in a humidified chamber. The cells were treated with DMSO (control), 10ng/ml IL-1β alone or 2.5, 5 or 10μM tomatidine pretreatment for 2 h followed by 10ng/ml IL-1β for 24h. Then, the total cellular proteins were extracted using the RIPA buffer containing 1% protease inhibitor and 1% phosphatase inhibitor (Boster, Wuhan, China). The concentration of total protein lysates was measured using the bicinchoninic acid (BCA) protein assay kit (Boster, Wuhan, China). Equal amounts of protein samples were separated on a 12% polyacrylamide gel, transferred onto 0.45μm polyvinylidene difluoride (PVDF) membranes (Millipore, Billerica, MA), andblocked with 5% BSA in Tris-buffered Saline-Tween solution (TBST) for 1h at room temperature. Then, the membranes were incubated overnight at 4°C with the primary antibodies against iNOS, MMP13, and GAPDH at 1:500 dilution, and antibodies against COX-2, MMP1, MMP3, ADAMTS-5, phospho-P38, P38, phospho-ERK, ERK, phospho-JNK, JNK, phospho-P65 and P65 at 1:1000 dilution. Then, after washing with TBST, the membranes were incubated with theHRP-conjugated secondary antibody for 1h at room temperature. The blots were developed with theEnhanced Chemiluminescence (ECL) reagent (ECL; Boster, Wuhan, China), and the protein bands were quantified using theBio-Rad Image Lab 5.0 system(Bio-Rad, USA).

### Immunofluorescence staining

The primary chondrocytes were seeded in a 24-well plate and treated with 10ng/ml IL-1β alone or in combination with 10μM tomatidine. The cells were then fixed with 4% paraformaldehyde for 10min, permeated with 0.1% Triton X-100 for 10min, and blocked with 1% BSA for 30min. Subsequently, the cells were incubated overnight at 4 °C with antibodies against collagen-II, aggrecan, and P65 at 1:200 dilutions. Then, after washing thrice with PBS, the cells were incubated with Cy3-conjugated goat anti-rabbit or Cy3- conjugated goat anti-rat secondary antibodies at a 1:75 dilution for 1h. The cells were incubated with 4–6-Diamidino-2-phenylindole (DAPI) for 10 min to stain the nuclei, and examined using a automated EVOS fluorescence microscope (Life Technologies, USA).

### Animal experiments and histological analysis

The animal protocol was approved by the Ethics and Animal Research Committee of Huazhong University of Science and Technology. We purchased male Sprague-Dawley rats weighing 250-300g from the Experimental Animal Center of Tongji Medical College (Wuhan, China) and induced OA as described previously [37]. The animals (n=5/group) were randomly divided into blank control, OA, and experimental groups. OA pathogenesis was induced by surgery involving anterior cruciate ligament transaction and partial medial meniscectomy [37]. The experimental group rats were fed with 25 mg/ Kg/ day tomatidine as previously described [38]. After 12 weeks, the animals were sacrificed, the tibiofemoral joints were collected, fixed with 4% paraformaldehyde for 24h, and decalcified by incubation in 10% EDTA for 20 days. Then, the specimens were embedded in wax blocks, cut into 3-5μm thick sections, and stained with safranin O-fast green(S-O) and toluidine blue. The stained specimens from all 3 rat groups were scored according to the Osteoarthritis Research Society International (OARSI) recommendations and graded in a blinded manner. Another set of tibiofemoral joint sections from the 3 groups were incubated with antibodies against INOS and MMP3. The numbers of INOS and MMP3 positive cells were determined using the Image-J software.

### Micro-computed tomography (μ-CT)

We scanned the right knee joints of the rats using the μ-Computed Tomography system (Scanco Medical, Bassersdorf, Switzerland). We obtained 21.0μm resolution μ-CT images at 70kV and 113μA and generated 3D images using the CT system software. The same region of interest (ROI) was drawn on 100 consecutive slices of 2.1mm total thickness representing the subchondral bone at the tibial plateau and the values for bone parameters such as bone volume/tissue volume (BV/TV), trabecular numbers (Tb.N) and trabecular separation (Tb.Sp) were determined.

### Statistical analysis

All experiments were performed in triplicate and the data presented as means ± standard deviation (SD). All data were analyzed using the Shapiro-Wilk test for normal distribution and Levene’s test for homogeneity of variances. The statistical differences between normally distributed groups were analyzed by one-way analysis of variance (ANOVA) and Student’s t-test. The nonparametric data (OARSI scores) were compared using the Kruskal-Wallis H test. P-value < 0.05 was considered statistically significant.
