# Correction for: Tomatidine suppresses inflammation in primary articular chondrocytes and attenuates cartilage degradation in osteoarthritic rats

**DOI:** 10.18632/aging.203892

**Published:** 2022-02-15

**Authors:** Xiangyu Chu, Tao Yu, Xiaojian Huang, Yang Xi, Bowei Ni, Rui Zhang, Hongbo You

**Affiliations:** 1 Department of Orthopedics, Tongji Hospital, Tongji Medical College, Huazhong University of Science and Technology, Wuha, Hubei, 430030, China; 2 Department of Orthopedic Surgery, Tongji Hospital, Tongji University School of Medicine, Shanghai, 200065, China

Original article: Aging. 2020; 12:12799–12811. https://doi.org/10.18632/aging.103222

**This article has been corrected:** The authors noticed errors in **Figure 9AB**. As a result of misfiling the data, some of the images for the Control group were mixed up with images for the OA+Td group during preparation of the figure. To correct the errors, in panel **9A** the toluidine blue- and safranin O-fast green (S-O)-stained images for the Control and OA+Td groups were replaced with representative images from the original sets of experiments. In panel **9B**, the image showing staining for INOS in the Control group and the immunohistochemistry images of MMP3 staining were replaced with the correct images for the Control and OA groups. All images were derived from the initial sets of experiments. The authors stated that these alterations do not affect the results or conclusions of this work and apologized for any inconvenience caused.

New **Figure 9** is presented below.

**Figure 9:**
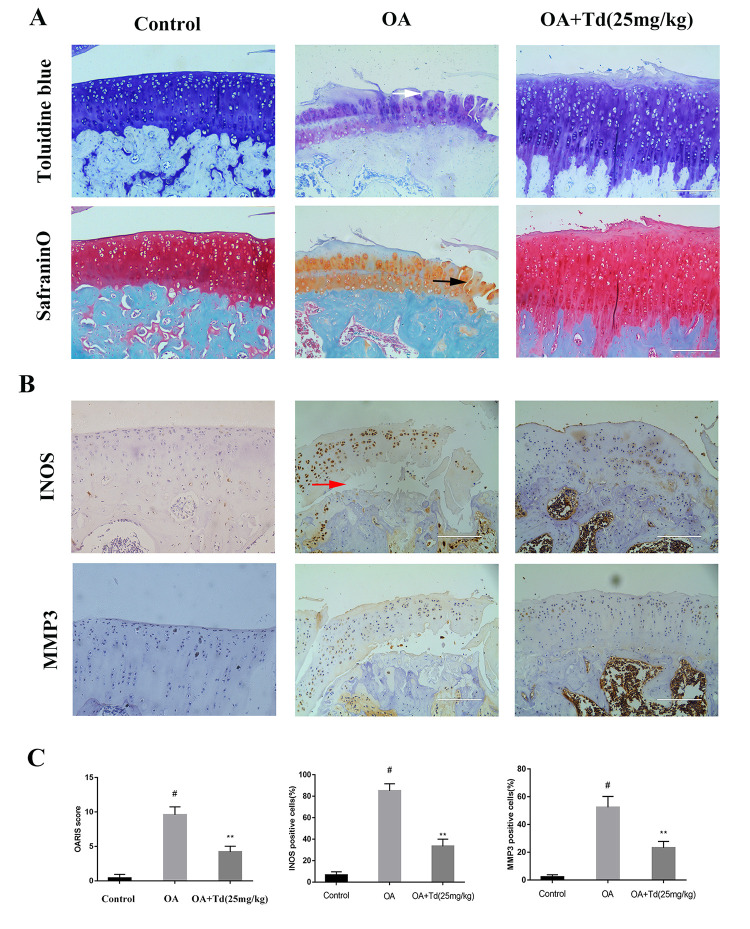
**Tomatidine ameliorates OA progression in the rat OA model. Sprague Dawley rats (n=5/group) were randomly divided into blank control, OA, and experimental groups.** The experimental group rats were fed a diet that included 25mg/kg/day tomatidine. The rats were maintained in these groups for 12 weeks and then euthanized and their tibiofemoral joints were obtained and processed. (**A**) Representative images show safranin O-fast green(S-O) and toluidine blue stained sections of tibiofemoral joints from blank control, OA and experimental group rats. The vertical fissures (black arrow), surface discontinuity (white arrow) and delamination (red arrow) are indicated in the relevant samples as shown. (**B**) Representative immunohistochemical stained images show the expression of iNOS and MMP3 proteins in tibiofemoral joint sections from blank control, OA and experimental group rats. (**C**) The OA grades of blank control, OA and experimental group rats at 12 weeks according to the Osteoarthritis Research Society International (OARSI) scores are shown. The scoring was performed in a blinded manner. The iNOS and MMP3 positive cells were counted in each tibiofemoral joint section from blank control, OA and experimental group rats and quantified using the Image-J software. #p < 0.05 compared with the control group. *p < 0.05 and **p < 0.01 compared with the OA group.

